# Novel functions of Fanconi anemia proteins in selective autophagy and inflammation

**DOI:** 10.18632/oncotarget.10970

**Published:** 2016-07-30

**Authors:** Rhea Sumpter, Beth Levine

**Affiliations:** Department of Internal Medicine, Center for Autophagy Research, University of Texas Southwestern Medical Center and Howard Hughes Medical Institute, Dallas, TX, USA

**Keywords:** Fanconi anemia, mitophagy, inflammasome, BRCA, innate immunity

Loss-of-function mutations in 19 Fanconi anemia (FA) pathway genes result in a variety of clinical manifestations such as birth defects, cognitive impairment, bone marrow failure (in up to 90% of FA patients within the first two decades of life), increased cancer risk, and accelerated aging [1]. Following DNA damage, proteins of the FA pathway act in a complex cascade to repair interstrand crosslinks (ICLs), which are caused by reactive oxygen species (ROS) or exposure to reactive aldehydes [2]. Although FA is a rare disease, somatic mutations or epigenetic silencing of several FA genes are found commonly in cancers in the general population (e.g. breast, ovarian, and pancreatic cancers). Furthermore, monoallelic germline mutations in at least five genes in the FA pathway, *FANCD1/BRCA2*, *FANCS/BRCA1*, *BRIP1/FANCJ*, *PALB2/FANCN*, and *RAD51C/FANCO* are associated with the development of cancer.

Selective autophagy is a homeostatic “cellular housekeeping” pathway in which unwanted cytoplasmic cargos are targeted for lysosomal destruction. We discovered that Fanconi anemia genes represent a new class of selective autophagy factors [3, 4]. Our findings may help to explain some of the pathophysiological features of FA, particularly in regard to “environmental factors” [1] that modulate FA disease progression, and may lead to the identification of novel therapeutic targets for FA.

**Figure 1:**
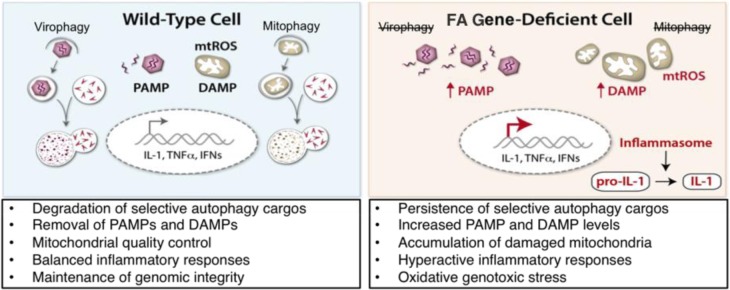
Pathophysiological consequences of defective FA gene-mediated selective autophagy In a wild-type cells (left panels), viruses and damaged mitochondria are targeted by selective autophagy. This process removes pro-inflammatory pathogenassociated molecular patterns (PAMPs; e.g. viral nucleic acids) and danger associated molecular patterns (DAMPs; e.g. mitochondrial (mt) ROS). Defective clearance of selective autophagy substrates in FA gene-deficient cells (right panels) results in persistence of PAMPs and DAMPs, increased inflammasome activation, and oxidative genotoxic stress.

We found that, while *Fancc* is dispensable for starvation-induced general macroautophagy, it is required for virophagy of two genetically unrelated neuronotropic viruses, Sindbis virus and herpes simplex virus type 1, and for host defense *in vivo* against lethal CNS infection with these viruses. *FANCC* is also required for Parkin-mediated mitophagy *in vitro* and for mitochondrial quality control *in vivo*. Thus, *FANCC* is required for two distinct forms of selective autophagy: virophagy and mitophagy (Figure 1).

Rather than playing an indirect role in selective autophagy, FANCC is likely to function as an adaptor protein in both virophagy and mitophagy. FANCC is contained within membrane-bound autophagolysosomal structures together with Sindbis viral nucleocapsids and (similar to previously identified virophagy factors p62 [5] and SMURF1 [4]) FANCC interacts biochemically with Sindbis virus capsid protein. FANCC also interacts biochemically with Parkin and colocalizes with damaged mitochondria in a Parkin-dependent manner. Thus, FANCC interacts with seemingly unrelated cargos (viral nucleocapsids and damaged mitochondria) to enable their targeting by the selective autophagy pathway.

Although other DNA repair pathway proteins (e.g. XPA [6]) have been implicated in mitophagy, it is unclear whether their roles in mitophagy can be segregated from their functions in DNA repair and general macroautophagy. We found that a naturally occurring hypomorphic mutant of FANCC (c.67delG) completely fails to complement DNA repair but is fully competent for mitophagy [3]. This observation may provide insight into the importance of FANCC-mediated selective autophagy in ameliorating the disease course of FA patients, as patients with FANCC c.67delG mutations are often phenotypically normal and have more mild disease than patients with null mutations in *FANCC* [1]. FANCC represents the first DNA repair pathway protein known to possess a DNA repair- and general macroautophagy-independent role in mitophagy.

Elevation of inflammatory markers and hypersusceptibility to proinflammatory cytokineinduced cell death is a phenotype associated with FA gene mutations [1, 2]. Damaged mitochondria potentiate proinflammatory signaling pathways, including the inflammasome, through their production of ROS [7]. Bacterial lipopolysaccharide (LPS)-induced mitophagy is defective in *Fancc^−/−^* primary BMDMs, resulting in elevated mitochondrial (mt)ROS levels, inflammasome hyperactivation, and elevated IL-1β secretion (Figure 1), which is attenuated in the presence of a mtROS scavenger. Antagonists of IL-1β signaling have been successful in clinical use for the treatment of several autoinflammatory diseases. Thus, based on our results, we speculate that therapeutic strategies targeting IL-1β signaling and/or inflammasome hyperactivation may prove effective for diseases associated with FA pathway gene mutations.

Importantly, we found that additional FA pathway proteins, including some with no known direct cytoplasmic function (i.e. FANCD2, FANCS/BRCA1, and FANCD1/BRCA2), are also required for mitophagy. The precise mechanism(s) by which FA proteins function in selective autophagy and the extent to which defects in FA protein-mediated selective autophagy contribute to diseases associated with FA gene mutations remain to be determined. However, our results provide surprising and unexpected new avenues for investigation into the causes of - and treatments for - FA and FA gene-associated diseases.

Our discovery of a role for FA proteins in mitophagy may have especially significant implications for understanding FA gene-associated diseases. The dual functions of FA proteins in mitophagy and DNA repair are likely to be teleologically linked, acting in cytoplasmic surveillance for damaged (ROS-producing) mitochondria and in the nuclear repair of ROS-inflicted ICLs. Since mitochondria are the largest source of endogenous ROS, failure of FA protein-mediated mitophagy presumably leads to the persistence of oncogenic, cell death-inducing, or senescence-associated oxidative DNA damage (Figure 1). We propose a FA gene-associated disease paradigm in which defective FA protein-mediated mitophagy results in the accumulation of mtROS, driving aberrant proinflammatory signaling and irreparable genotoxic stress, ultimately leading to phenotypes such as bone marrow failure, cancers, and aging associated with mutations in FA pathway genes.
